# Intraoperative device closure of perimembranous ventricular septal defects in the young children under transthoracic echocardiographic guidance; initial experience

**DOI:** 10.1186/1749-8090-6-166

**Published:** 2011-12-28

**Authors:** Hua Cao, Qiang Chen, Gui-Can Zhang, Liang-Wan Chen, Qian-Zhen Li, Zhi-Huang Qiu

**Affiliations:** 1 Department of Cardiovascular Surgery, Union Hospital, Fujian Medical University, Fuzhou, 350001, P. R. China

**Keywords:** CHD, septal defects, Cardiac intervention, Surgery

## Abstract

**Objectives:**

This study aimed to assess the safety and feasibility of intraoperative device closure of perimembranous ventricular septal defects (VSD) in young children guided by transthoracic echocardiography (TTE).

**Methods:**

We enrolled 18 patients from our hospital to participate in the study from June 2011 to September 2011. A minimal inferior median incision was performed after full evaluation of the perimembranous VSD by real-time TTE, and a domestically made device was inserted to occlude the perimembranous VSD. The proper size of the device was determined by means of transthoracic echocardiographic analysis.

**Results:**

Implantation was ultimately successful in 16 patients using TTE guidance. In these cases, the complete closure rate immediately following the operation and on subsequent follow-up was 100%. Symmetric devices were used in 14 patients, and asymmetric devices were used in two patients. Two patient were transformed to surgical treatment, one for significant residual shunting, and the other for unsuccessful wire penetration of the VSD. The follow-up periods were less than nine months, and only one patient had mild aortic regurgitation. There were no instances of residual shunt, noticeable aortic regurgitation, significant arrhythmia, thrombosis, or device failure.

**Conclusions:**

Minimally invasive transthoracic device closure of perimembranous VSDs is safe and feasible, using a domestically made device under transthoracic echocardiographic guidance, without the need for cardiopulmonary bypass. This technique should be considered an acceptable alternative to surgery or device closure guided by transesophageal echocardiography in selected young children. However, a long-term evaluation of outcomes is necessary.

## Introduction

Ventricular septal defect (VSD) is one of the most common congenital cardiac defects. The perimembranous VSD accounts for 70% of autopsy findings in surgical series, and is situated in the area wedged between the tricuspid and aortic valves. A significant proportion of these defects require closure [1]. Transcatheter closure and surgical repair are reliably achieved with no mortality and minimal morbidity [2-4]. As an alternative to these closure methods, we employed an intraoperative device and minimally invasive surgery to close perimembranous VSDs without cardiopulmonary bypass (CPB) [5-7]. This minimally invasive treatment offers a good cosmetic effect and is more acceptable to patients. We have achieved high technical success and good acute outcomes. Meanwhile, the procedure requires no advanced or expensive equipment, and the cost is acceptable in low-income nations. There are many reports on the use of transesophageal echocardiography (TEE) for transcatheter guidance or periventricular placement of a VSD closure device [3,4,6]. To our knowledge, there are no other published reports on the role of TTE in guidance of intraoperative device closure of perimembranous VSDs. TTE also provides an accurate and noninvasive definition of the perimembranous VSD anatomy using subcostal views, especially in young children. Therefore, we used the subcostal window to provide an alternative to guide the intraoperative device placement. This approach is easy to learn and manipulate. The aim of this study was to evaluate the safety and feasibility of intraoperative device closure of the perimembranous VSD in young children, under transthoracic echocardiographic guidance.

## Matreials and methods

This study was approved by the ethics committee of our university and was conducted in accordance with the tenets of the Declaration of Helsinki. Written informed consent was obtained from the parents of the patients.

### Devices

**Figure 1:**
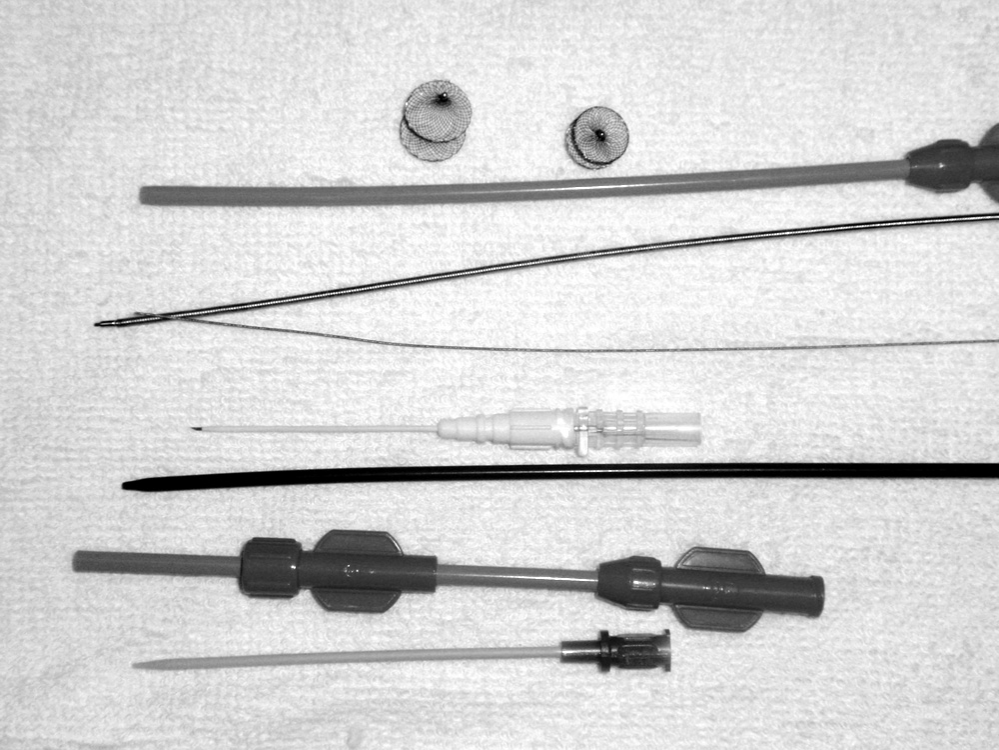
**The asymmetric device, the symmetric device, and the entire delivery system**.

The entire delivery system consisted of a trocar, guide wire, dilator and delivery sheath, and a loading sheath made of polyethylene and acrylonitrile butadiene styrene and disinfected with ethylene oxide. The device was made by Lifetech Scientific (Shenzhen) Co, LTD of China. The VSD occluder was a self-expandable, double-disk device. There are two types of occluder, symmetric and asymmetric, that differ in their left ventricular dish style. On the left ventricular side of asymmetric device, the aortic end of the disk is 1 mm wider than the waist, so as to avoid impinging the aortic valve. The other side is 5 to 6 mm wider than the waist and has a platinum marker to guide device orientation. The symmetric device has a left ventricular disk that is 2 mm larger than the waist. The right ventricular disk is 2 mm larger than the waist in both types of occluders (Figure 1). The size of the occluders is based on the waist diameter, which ranged from 6 to 14 mm in 1-mm increments. The occluders were implanted with a 6-9 F delivery sheath [6].

### Patients

In the period between June 2011 and September 2011, 18 young children with perimembranous VSDs received intraoperative, minimally invasive device closure in our hospital. VSD was detected using TTE performed initially using standard transthoracic imaging with color and Doppler interrogation from subxiphoid, apical, and parasternal views before the closure procedure. The patients ranged in age from 2 to 36 months (mean ± standard deviation, 12.0 ± 7.4 months) and weighed from 5 to 18 kg (mean = 10.2 ± 3.6 kg). For inclusion in the study, (1) patients had to have perimembranous VSDs, (2) a distance of over 2 mm rims of the VSD to nearby major cardiac structures, such as the aortic, tricuspid, and pulmonary valves, and (3) no aortic regurgitation. The procedure was used in patients weighing more than 5 kg, or having VSD of less than 10 mm in diameter, and a subaortic rim greater than 2 mm. Individuals with VSDs of other sizes or locations or exhibiting coexisting cardiac anomalies were excluded from our study. Other inclusion criteria for intraoperative device closure were the same as those used for surgical closure or transcatheter closure, which included hemodynamically significant L-R shunts and/or significant chamber enlargement, with or without mild-moderate pulmonary hypertension despite medical therapy, and a history of infective endocarditis. Patients were given routine examinations, including a standard electrocardiogram, chest X-ray, and blood tests. All patients had normal sinus rhythm and normal left ventricular (LV) systolic function. Eight patients were symptomatic for respiratory infection, palpitations, or exercise intolerance. Defect diameters as measured by TTE ranged from 5 to 9 mm (mean = 6.5 ± 1.0 mm). Six patients exhibited moderate pulmonary hypertension (pulmonary arterial pressure > 40 mmHg).

### Protocol

TTE was used to confirm the diagnosis of the isolated perimembranous VSD in patients and to assess the circumferential margins for closure from subcostal, apical, and parasternal views, especially from the edge to the aortic valve. VSD diameter was measured by TTE before the operation, using two-dimensional imaging and color flow Doppler on long- and short-axis views. The choice of occluder size was usually based on the largest size of the defect on the TTE color Doppler image and was basis on the maximum TTE-measured diameter plus 0-2 mm. The asymmetric device allowed for a margin of 0-2 mm in excess of the waist diameter, while the symmetric device is 2 mm in excess. When the defect was aneurysmal or fenestrated, the maximum size of the mouth of the aneurysm (true VSD) was recorded, as well as the exit point of the main defect into the right ventricle (RV).

**Figure 2:**
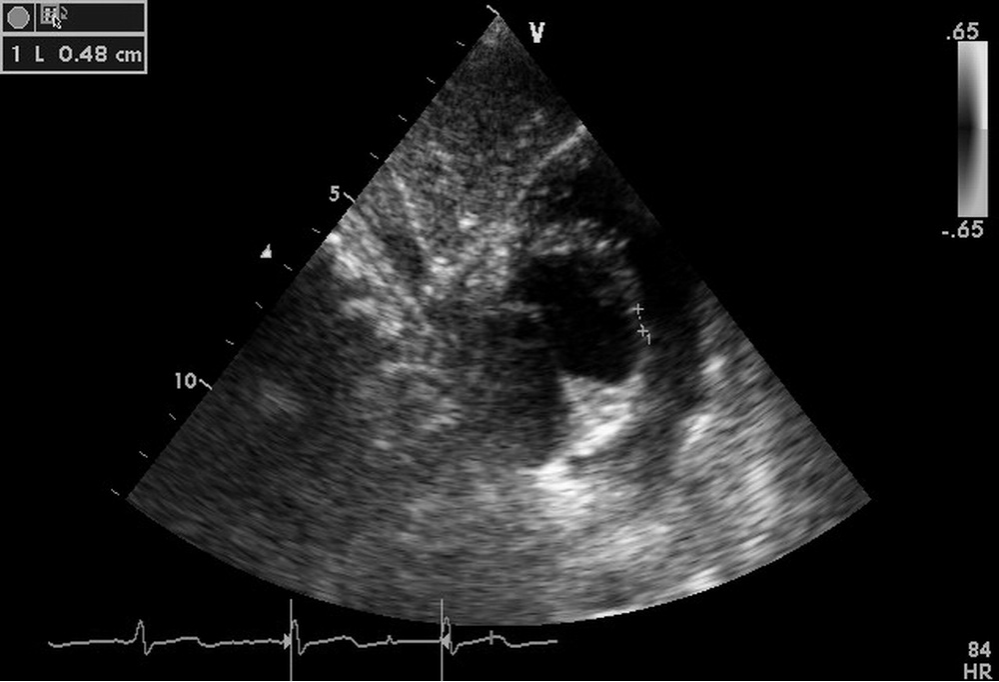
**The image of the perimembranous VSD showed by TTE subcostal views**.

**Figure 3:**
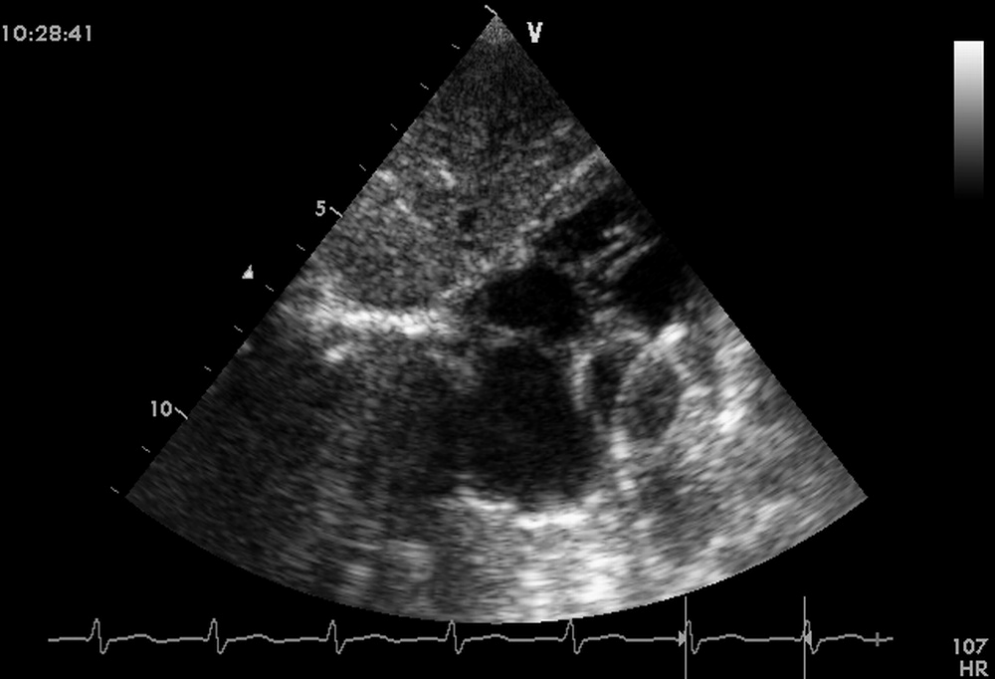
**A floppy wire positioned from the right ventricle free wall into the left ventricle cavity across the VSD**.

**Figure 4:**
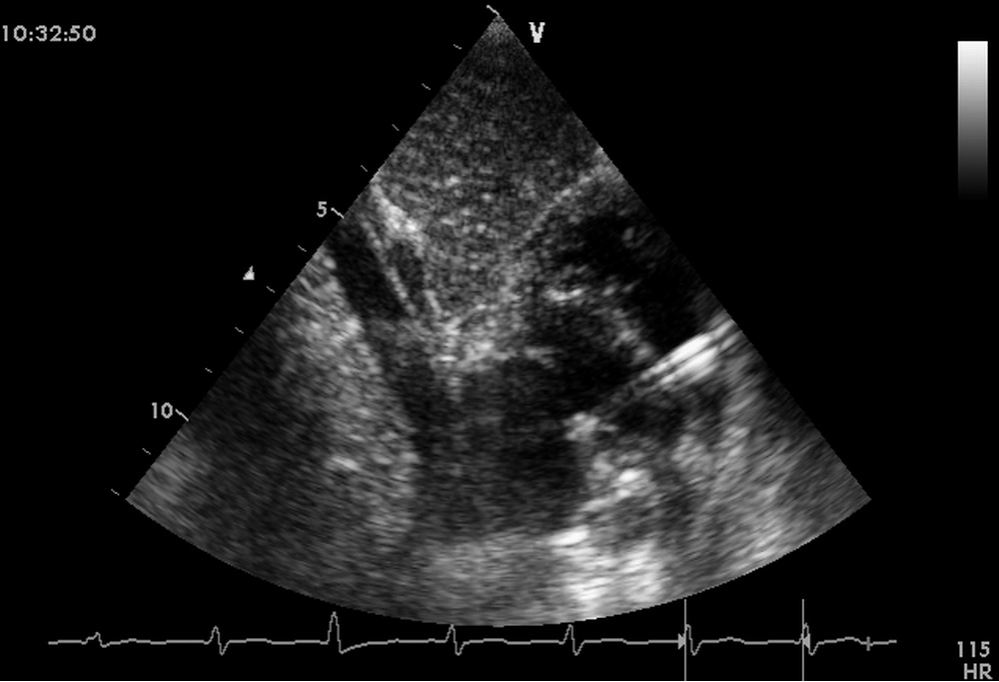
**A sheath was advanced over the wire into the left ventricle across the VSD**.

**Figure 5:**
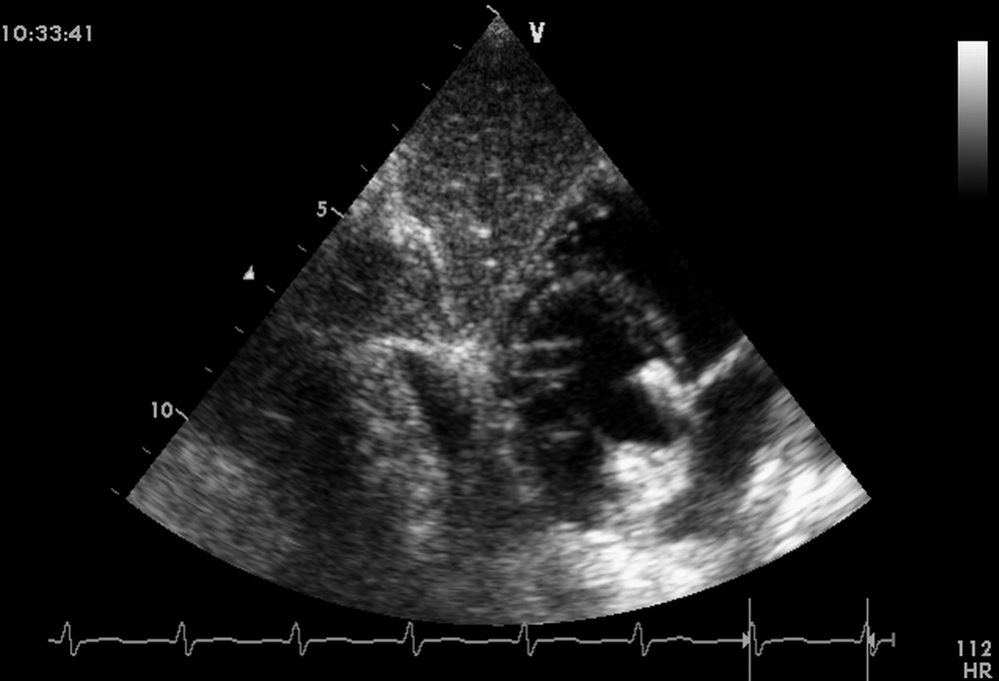
**The left disc was deployed**.

**Figure 6:**
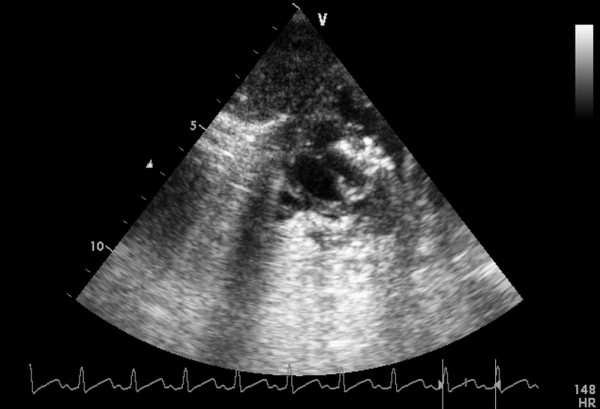
**Final image showed after an asymmetric device was deployed**.

**Figure 7:**
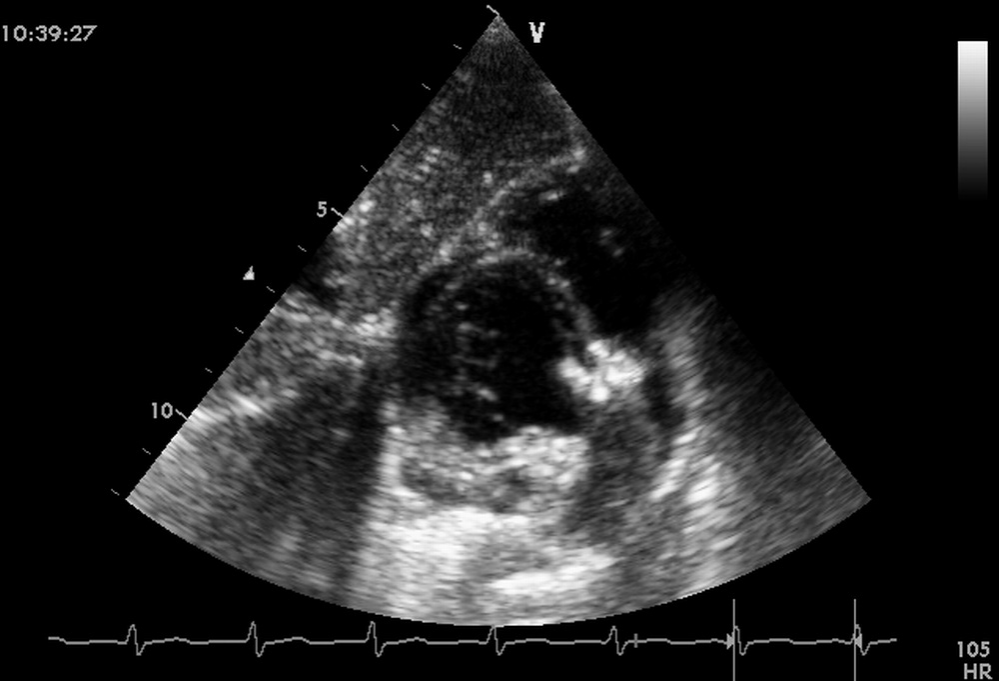
**Final image showed after a symmetric device was deployed**.

General anesthesia was administered to patients, after which they were placed in a supine position and draped for exposure of the entire chest with a probe placed below the xiphoid process to guide the whole procedure (Figure 2). An inferior median sternotomy was performed (about 3 cm). A small rib spreader was used in this manipulation incision to facilitate instrumentation. The pericardium was opened and suspended to expose the right ventricle. On the free wall of the right ventricle, two parallel 5/0 or 4/0 Prolene sutures of approximately 8 mm in diameter were stitched. Heparin (1 mg/kg body weight) was administered intravenously, and the activated clotting time was monitored to be greater than 250 seconds. A modified short angiocatheter was passed into the right ventricle through the free wall, and the needle was removed. A floppy wire was advanced through the angiocatheter and aimed toward the perimembranous VSD. The VSD was crossed, and the wire was advanced through the defect into the left ventricle (Figure 3). The dilator was removed, and an appropriately sized delivery sheath was advanced over the wire into the left ventricle. The wire and the dilator were removed, and the sheath was allowed to back bleed to ensure there was no entrapment of air (Figure 4). The VSD occluder was immersed in saline, and screwed onto the delivery cable. The device was loaded with the help of a loading sheath. The loading sheath was introduced into the delivery sheath, and the occluder was advanced to the tip of the sheath. The sheath was pulled back gently to leave the tip in the left ventricle. Under TTE guidance, the left disc was deployed, and the sheath was pulled back slowly until the left disc approximated the ventricular septum (Figure 5). To implant the asymmetric device, it was rotated gently so that the platinum marker of the distal disk pointed downward and the flat part of the disc was not directly under the aortic valve (Figure 6). The waist and the right disc of the occluding device were deployed while maintaining moderate traction on the delivery cable (Figure 7). When TTE evaluation showed there was no significantly residual shunt and no significant aortic or tricuspid valve regurgitation, the occluder was released by rotating the delivery cable counterclockwise. The sheath and the delivery cable were withdrawn with the suture tied snugly. Routine chest closure with placement of a drainage tube were performed. Oral dipyridamole or aspirin were prescribed to be taken for 3-6 months as an anticoagulant [5-7].

### Statistical Analysis

Continuous data were presented as means ± standard deviation and range.

## Results

Occluder delivery was successful in 16 patients using TTE guidance. The other two patients were transformed to surgical closure, with significant residual shunting resulting in occluder removal in one patient, and failure to establish the occluder conveying rail in another patient. In the 16 device-closure patients, the size of the implanted occluder ranged from 6 to10 mm (8.1 ± 0.9 mm), and the diameter of the sheath was 6-9 F. Symmetric devices were used in 14 patients, and asymmetric devices were used in two patients. The duration of the procedure was 40 to 60 minutes (45 ± 5.5 minutes). Patients were required to remain in the intensive care unit for 12 to 24 hours (15.5 ± 1.4 hours), and to remain in the hospital for 3 to 6 days (3.5 ± 1.3 days). In those for whom occluder delivery was successful, the overall immediate complete closure rate was 100%. All patients were extubated within 12 hours and were discharged within about 3 to 4 days after operation.

Minor complications were encountered in two patient, including transient arrhythmia during device implantation in one patient. Temporary sinus bradycardia was observed immediately in this patient, but a normal rhythm recovered spontaneously. Mild aortic regurgitation was found in one other patient, who was followed up for 6 months without significant symptoms or aortic regurgitation deterioration. No episodes of cardiac block, thromboembolism, or device displacement were observed in the follow-up period of hospitalization.

The total follow-up periods were less than 9 months in duration and were to be continued. Out-patient follow-up was by functional, TTE, and ECG assessment. In all symptomatic patients, symptoms were either resolved totally or improved significantly. In those patients with mild pulmonary hypertension, symptoms decreased significantly as evaluated by tricuspid regurgitation jet. The complete closure rate was 100% during the follow-up. There were no episodes of hydrothorax, endocarditis, thromboembolism, device disruption or failure, aortic valves distortion, complete atrioventricular block, or permanent rhythm disturbances. Chest incisions were minor and cosmetic.

## Disscussion

Ventricular septal defect (VSD) is one of the most common congenital cardiac defects. Although it has been established that both surgical and transcatheter closure of VSDs are very safe, with negligible mortality rates [2-4], these two methods still have some shortcomings. They continue to be associated with large midline sternotomy and cardiopulmonary bypass, resulting in longer hospital stays after closure surgery. Meanwhile, percutaneous approaches may cause potential vascular injury, patients must be exposed to X-rays, and the techniques involve expensive equipment and high costs. We used a hybrid method as reported by Xing Quansheng and his colleagues to close perimembranous VSDs [5], which features intraoperative device closure and a minimally invasive procedure. The use of TEE to guide transcatheter or periventricular device closure placement is well known and has been associated with good results [3-5]. In our series, we used TTE to guide the intraoperative device closure of the perimembranous VSD, which can provided a useful subcostal window to finish the procedure in the young children.

We limited the device closure to young patients weighing more than 5 kg, with perimembranous VSDs of less than 10 mm diameter with a sufficient subaortic rim. The inferior median sternotomy offered an operative field and allowed the cardiac surgeons to do better with traditional surgical techniques. The length of incision could be limited to 3 cm, and the procedure time could be significantly shortened to 40 to 60 minutes, owing to the avoidance of CPB. Moreover, our minimal incision was easy to extend for conversion to a regular open-heart procedure in the event of failure with intraoperative device closure, without the need for additional incisions or rearranging the operation from the catheterization laboratory to the operating room. This helped to ensure the safety of the procedure. Consequently, patients experienced less trauma, shorter operation times and hospital stays, and faster recoveries than those receiving surgical repair. Our method provides a perpendicular angle to the perimembranous ventricular septum, offering greater ease in guiding the wire through the VSD and deploying the occluder into the defect compared with transcatheter device closure. The key point for the perimembranous VSD device closure was the choice of the location for the placement of the occluding device. Choosing patients with a sufficient subaortic rim can guarantee the aortic valve will function from the influence of the occluder. It was important to choose an occluder of the proper size as measured by TTE, because one that is too large will lead to aortic regurgitation, and one that is too small will lead to residual shunting. In some patients, the symmetric device may interfere with the subaortic rim and lead to aortic regurgitation, and in these instances an asymmetric device can be applied. The special design of the asymmetric device allowed us to rotate and move the sheath by hand to orient the platinum marker of the distal left disk downward and position the flat part of the left disc so that it was not directly under the aortic valve. The 1-mm superior rim of the left ventricular disk should avoid contact with the aortic valve, while the larger 5-6 mm inferior rim with the platinum marker clasps the muscular septum. It was necessary to observe the location of the occluder by TTE for about 10 to 20 minutes after release before the sheath and the delivery cable were withdrawn. This allowed us to retrieve the occluder and try again if the closure results were not satisfactory.

In our study, device closure was successful in 16 patients. In two other patients that were referred for surgical closure, we were not able to achieve a suitable or stable occluder position, resulting in significant residual shunting in one case and a floppy wire spanning the defect in the other case. According to our experience, trivial or small residual shunts could be ignored immediately after the release of device, since they usually disappeared spontaneously during follow-up period. After several weeks, endothelialization will occur on the surface of the device, and neointima will form to fully close any residual shunting [6]. In the other case, the lack of success leaving a floppy wire across the defect was due to the small size VSD. After a second attempt we still couldn't locate the VSD, and the procedure was discontinued. During the surgical closure, we found this perimembranous VSD was within about 3 mm of a ventricular septal aneurysm. Minor complications were encountered in two patients. Temporary sinus bradycardia, which easily recovered spontaneously, was observed in the course of device implantation in one patient.

The only serious arrhythmic problem associated with perimembranous VSD closure is the occurrence of complete atrioventricular block (AVB), which can occur days, weeks, or even months later. Sporadic studies have reported the incidence of transient complete AVBs in up to 1-5% of patients undergoing transcatheter device closure of perimembranous VSDs [8-12]. Fortunately, we did not encounter complete AVB in our study.

Compared with transcatheter closure of the perimembranous VSD, our approach provided a perpendicular angle to the perimembranous ventricular septum. This allows the surgeon to easily guide the wire through the VSD and deploy the occluder into the defect. The chance of conduction system injury from mechanical trauma/compression by the delivery system or device seemed to be small in our series. Careful manipulation of devices while crossing the defect is important and can reduce the occurrence of arrhythmia. A suitable size and position of the device must be verified carefully using TTE, because using an oversized device or dislodgement of a too-small device may increase the risk of arrhythmia. In our experience, the device selected should be the same size or 1 to 2 mm larger than the VSD, as measured by TTE. We recommend abandoning the procedure if complete AVB develops.

Mild aortic regurgitation was found only in one patient, who was followed up for six months without significant symptoms or aortic regurgitation deterioration. The estimated incidence of aortic regurgitation following transcatheter closure ranges from 3.4 to 13.6%, leading to complications that require urgent surgical retrieval of the device [13]. Significant aortic regurgitation following device occlusion may be due to impingement of the left ventricular disc on the leaflets of the aortic valve. The specially designed superior rim of the asymmetric device can maximum reduce the influence of the aortic valve functions. We also suggest those patients with aortic regurgitation during the device closure procedure should turn to surgical closure.

In those for whom the procedure was successful, the closure rate were very impressive. Although there were no episodes of cardiac block, thromboembolism, or device displacement in the follow-up period, our follow-ups were limited. More attention should be paid to the routine follow-up.

It is well known that imaging of the perimembranous VSD by TEE plays an essential role in the device closure of ventricular septal defects. TEE is superior to any other method for the measurement of rims and dimension of VSD [14,15]. Our study demonstrates that TTE may be employed for definitive assessment of the perimembranous VSD, selection of patients for device closure, and guidance of intraoperative periventricular device placement in young children. This is because subcostal views provide relatively clear images of the perimembranous VSD anatomy in young children. The resolution and image quality of TTE is not as good as TEE, but as we stated before, in a special subset of patients the result of TTE-guidance to close the perimembranous VSD were the same as that with TEE-guidance. This determines the accuracy of examination results and affects the procedure of VSD closure guidance. The deficiency of aortic rim is considered a risk factor for unsuccessful closure and a significant predictor for residual leakage [6], so we did not include patients with aortic rim deficiencies or aortic regurgitation in this study. Moreover, with careful manipulation, TTE also provides an accurate distance from the edge of the VSD to the aortic valve. Fortunately, the majority of the perimembranous VSD patients in this study had sufficient aortic rims, so the symmetric devices were mainly applied in these circumstances. We would also emphasize that TTE-guided perimembranous VSD closure was only used with care by experienced surgeons and echocardiographers. Concerning the use of TEE in infants, there may still be a risk of complications such as gastroesophageal injury and respiratory compression [16]. We might also still approve of using TEE to guide the VSD device closure as the first choice, and do not advocate the use of TTE to substitute completely for TEE. Our initial study just shows that TTE by providing subcostal views also offers a feasible means of guiding the VSD device placement and could be as an alternative. With increasing experience we hope that performing perimembranous VSD closure procedures under TTE guidance can reduce procedure time and also provide increased patient comfort, and that it may reduce the need for TEE.

Like any retrospective study, there is bias associated with data collection and the incomplete data for some patients. As a result of the mentioned 16 successful device closure cases, the conclusions were limited to our own experience. Meanwhile, the average follow-up was very short, and longer follow-up periods are needed in future research. This study was limited to one institution, and other institutions may find different results. In our study, the occluder was less expensive than the Amplatzer occluder. The technique did not require an expensive X-ray machine and was relatively easy to master. This method should be the treatment of choice for the effective treatment of perimembranous VSDs.

In conclusion, our study demonstrates that intraoperative device closure of perimembranous VSDs using TTE-guidance is safe and feasible. It has the advantages of yielding cosmetic results and leaving less trauma than surgical closure. Our initial study shows that TTE-generated subcostal views can also to be used to as an alternative to guide VSD device placement in young children.

## Conflict of interests

The authors declare that they have no competing interests.

## Authors' contributions

HC collected the clinical data and performed the statistical analysis, participated in the operation and drafted the manuscript. QC participated in the operation and drafted the manuscript, revised and submitted the paper. LC and HC designed the study and performed the operation. QL and ZQ participated in the operation. GZ was the operator of echocardiography for intraoperation TTE surveillance and provided the ultrasound images. All authors read and approved the final manuscript.
